# Identification and Pathogenic Potential of Clinical *Bacillus* and *Paenibacillus* Isolates

**DOI:** 10.1371/journal.pone.0152831

**Published:** 2016-03-31

**Authors:** Francesco Celandroni, Sara Salvetti, Sokhna Aissatou Gueye, Diletta Mazzantini, Antonella Lupetti, Sonia Senesi, Emilia Ghelardi

**Affiliations:** 1 Department of Translational Research and New Technologies in Medicine and Surgery University of Pisa, Pisa, Italy; 2 Department of Biology University of Pisa, Pisa, Italy; 3 Interdepartmental Research Center Nutraceuticals and Food for Health, University of Pisa, Pisa, Italy; Indian Institute of Science, INDIA

## Abstract

The soil-related *Bacillus* and *Paenibacillus* species have increasingly been implicated in various human diseases. Nevertheless, their identification still poses problems in the clinical microbiology laboratory and, with the exception of *Bacillus anthracis* and *Bacillus cereus*, little is known on their pathogenicity for humans. In this study, we evaluated the use of matrix-assisted laser desorption—ionization time of flight mass spectrometry (MALDI-TOF MS) in the identification of clinical isolates of these genera and conducted genotypic and phenotypic analyses to highlight specific virulence properties. Seventy-five clinical isolates were subjected to biochemical and MALDI-TOF MS identification. 16S rDNA sequencing and supplemental tests were used to solve any discrepancies or failures in the identification results. MALDI-TOF MS significantly outperformed classical biochemical testing for correct species identification and no misidentification was obtained. One third of the collected strains belonged to the *B*. *cereus* species, but also *Bacillus pumilus* and *Bacillus subtilis* were isolated at high rate. Antimicrobial susceptibility testing showed that all the *B*. *cereus*, *B*. *licheniformis*, *B*. *simplex*, *B*. *mycoides*, *Paenibacillus glucanolyticus* and *Paenibacillus lautus* isolates are resistant to penicillin. The evaluation of toxin/enzyme secretion, toxin-encoding genes, motility, and biofilm formation revealed that *B*. *cereus* displays the highest virulence potential. However, although generally considered nonpathogenic, most of the other species were shown to swim, swarm, produce biofilms, and secrete proteases that can have a role in bacterial virulence. In conclusion, MALDI-TOF MS appears useful for fast and accurate identification of *Bacillus* and *Paenibacillus* strains whose virulence properties make them of increasing clinical relevance.

## Introduction

A variety of classic and emerging soil-related bacterial and fungal pathogens causes serious human disease that frequently presents in primary care settings. Members of the genus *Bacillus* and *Paenibacillus* (originally *Bacillus* group 3) [1] are large, gram-positive rod bacteria that are ubiquitous in the environment and often found in soil, air, water, and food. They form spores that are resistant to heat, cold, and common disinfectants, allowing them to survive on environmental surfaces for prolonged periods [2]. Very few species belonging to these genera are considered medically relevant so far. *Bacillus anthracis* is the etiological agent of the acute and often lethal disease anthrax and *Bacillus cereus*, commonly known to cause food-borne intoxications, also causes local and systemic infections [3]. The other species are generally perceived of little clinical significance and they are commonly considered as contaminants in clinical cultures. However, recent reports indicate that these organisms can be responsible for local or systemic infections in humans [4–11].

The pathogenicity of non-anthrax *Bacillus* species has been poorly investigated, except for *B*. *cereus*. The pathogenic potential of this bacterium has been related to the secretion of several virulence proteins [3, 12] and to motility factors, such as swimming and swarming [13]. The virulence proteins include several hemolysins, phospholipases, trimeric toxins (hemolysin BL, HBL; non-hemolytic enterotoxin, NHE), cytotoxin K (CytK) and proteases [3, 12, 14, 15]. HBL, NHE, and CytK act as tissue destructive/reactive proteins damaging the integrity of the plasma membrane of several cells [12]. In addition, *B*. *cereus* produces biofilms, which can play a major role in attachment to catheters [16, 17]. Some other *Bacillus* spp. were shown to contain DNA sequences encoding the HBL, CytK or NHE [18, 19] or to be able to produce biofilms [20, 21].

The identification of species in the genus *Bacillus* by classical methods is often difficult, due to similarities among closely related species that share a pattern of morphological, biochemical, and genetic characteristics. These unusual similarities are particularly evident among members of the *B*. *cereus sensu lato* group that comprises, other than *B*. *cereus* and *B*. *anthracis*, other species, such as *Bacillus thuringiensis* and *Bacillus mycoides* [22], showing almost identical 16S rRNA gene sequences and a high level of chromosomal synteny [23].

The use of matrix-assisted laser desorption—ionization time of flight mass spectrometry (MALDI-TOF MS) as a diagnostic technique for bacterial identification could prove quite useful in addressing the challenges associated with the identification of these organisms. This technology has been shown to successfully identify a wide range of clinically relevant bacteria and studies have also applied MALDI-TOF MS to some *Bacillus* [24–29] and *Paenibacillus* spp. [5, 30]. However, no comprehensive studies have evaluated the application of MALDI-TOF MS for the identification of clinical isolates of these genera.

The aim of the present study was to evaluate the use of MALDI-TOF MS for the identification of clinical *Bacillus* and *Paenibacillus* isolates and to investigate on their virulence potential by assessing hemolytic, phospholipase, and protease activities, motility and ability to form biofilms, as well as the presence of toxin encoding genes.

## Materials and Methods

### Clinical isolates

The clinical isolates tested in this study were collected in routine clinical workflow from specimens submitted to the Pisa University Hospital, Italy, over a two-year period (S1 Table). Multiple isolates from the same patient and body site were excluded. Cultures were processed per standard laboratory practices and, once pure culture was obtained on blood agar plates, strains were identified according to the operating procedures of our laboratory. This included microscopy of Gram-stained preparations and biochemical analysis using the API 50 CHB test kit according to the manufacturer’s instructions and the ATBPlus software (bioMérieux, Marcy l'Etoile, France). In parallel, bacteria from single colonies were used for MALDI-TOF MS analysis in a MALDI Biotyper Microflex LT mass spectrometer (Bruker Daltonik, Bremen, Germany). Failure to identify the organism, or any discrepancies between MALDI-TOF MS and biochemical identification, prompted 16S rRNA gene sequencing of the isolate.

The study was approved by the Ethical Committee Area Vasta Nord-Ovest, University of Pisa, and conducted in full accordance with the principles of the Declaration of Helsinki. Samples were taken as part of the standard patient care and used anonymously. For this type of study, no written informed consent was necessary.

### MALDI-TOF MS analysis

The isolates were tested in duplicate. A colony was directly spotted on the MALDI plate, and then overlaid with 1 μl of saturated α-cyano-4-hydroxycinnamic acid and air-dried. The loaded plate was then placed in the instrument according to the manufacturer’s instructions. The mass spectra were acquired within 10 minutes. The spectra were imported into the integrated MALDI Biotyper software (version 3.0) and analyzed by standard pattern matching with a default setting. A score of ≥ 2.00 indicated identification at the species level, a score from 1.70 to 1.99 indicated identification at the genus level, whereas any score under 1.70 meant no significant similarity of the obtained spectrum with any database entry. If the results were questionable, bacteria were retested after the standard protein extraction step employing formic acid and acetonitrile.

### 16S rRNA gene sequencing

Genomic DNA was extracted and purified as previously described [31]. The universal primers 27F (5’-GAGAGTTTGATCCTGGCTCAG-3’) and 1495R (5’-CTACGGCTACCTTGTTACGA-3’) were used for 16S rRNA gene amplification and sequencing (Eurofins MWG Operon, Germany). Sequences were compared with those contained in the Ribosomal Database Project. The isolates were identified according to the Clinical and Laboratory Standards Institute (CLSI) guidelines [32] by an identity score of 97% for genus and 99% for species.

### Supplemental testing and taxonomic resolution

The isolates identified by any of the methods as *B*. *cereus*, *B*. *thuringiensis*, or *B*. *mycoides* were analyzed for the presence of parasporal crystals, which are discriminative for *B*. *thuringiensis*. Sporulating cultures were propagated on sporulation medium and microscopically examined for the presence of the typical parasporal crystals in methanol-fixed preparations stained with 0.5% basic fuchsin [33]. The previously described *B*. *thuringiensis* RM1 strain was used as a positive control [33]. The same isolates were also analyzed for rhizoidal growth and inability to swim, which are discriminative for *B*. *mycoides* [34]. The *rpoB* gene sequences were amplified from the strains identified by 16S rDNA sequencing as *B*. *cereus*/*B*. *thuringiensis* using the primers *rpoB*1206 (5’-ATCGAAACGCC TGAAGGTCCAAACAT-3’) and *rpoB*R3202 (5’-ACACCCTTGTTACCGTGACGACC-3’) [35]. Amplification and sequencing reactions were performed as previously described by Ki and coworkers [35]. Sequences were compared with nucleotide sequences in the GenBank database using the BLAST search algorithm.

### Susceptibility testing

MICs to penicillin (PEN), ciprofloxacin (CIP), tetracycline (TET), tigecycline (TGC), and vancomycin (VCM) were determined by E-test (bioMérieux, Inc., Hazelwood, MO) on Mueller Hinton agar containing 5% blood and incubated at 35°C in air for 24h. *Staphylococcus aureus* ATCC 29213 was included as a control. The CLSI interpretative criteria for PEN (S: ≤ 0.12; R: ≥ 0.25), TET (S: ≤ 4; I: = 8; R: ≥ 16), and VCM (S: ≤ 4) against fastidious Gram positive bacilli were used [36]. The interpretative criteria described for *Bacillus* strains by Luna and colleagues [37], i.e. S: ≤ 1.0, R: ≥ 4 for CIP and S: ≤ 0.5 for TGC, were tentatively adopted.

### Motility and biofilm

Swimming and swarming motility were examined as previously described [38, 39]. Briefly, for swimming motility assays, 0.5 μl of an overnight culture (2×10^8^ cells ml^−1^) were spotted onto the center of TrM plates (1% tryptone, 0.5%, NaCl, 0.25% agar) and growth halo diameters were measured after a 6–8 h incubation at 37°C in a humidified chamber. Swimming motility was also confirmed under a phase-contrast microscope. Assays for swarming were initiated by spreading 50 μl of a culture (2×10^4^ cells ml^−1^) onto TrA plates (1% tryptone, 0.5%, NaCl, 0.7% agar) and incubating at 37°C in humidified chambers. Bacteria were tested for biofilm formation in Luria Bertani (LB) and, when biofilm was not formed in this medium, the assays were repeated in EPS, a low nutrient medium that contains 7 g of K_2_HPO_4_, 3 g of KH_2_PO_4_, 0.1 g of MgSO_4_ · 7H2O, 0.01 g of CaCl_2_, 0.001 g of FeSO_4_, 0.1 g of NaCl, 1 g of glucose, and 0.125 g of yeast extract (Difco) per liter [40]. Overnight cultures were adjusted to an optical density at 620 nm (OD_620_) of 0.01 in LB or EPS. Then, 2 ml were transferred to wells of polystyrene 24-well plates (Falcon/Becton Dickinson, Franklin Lakes, NJ), followed by incubation at 37°C and 50 rpm shaking for 8 h. The total growth (OD_620_) in each well was measured; planktonic bacteria were removed and the wells washed with distilled water and air-dried. Biofilms were stained with 2 ml of 0.3% crystal violet for 10 min, washed with distilled water, and air-dried. The crystal violet was solubilized with 2 ml of 70% ethanol and the optical density at 590 nm (OD_590_) was measured.

### Detection of virulence factors and toxin encoding genes

The bacterial ability to secrete hemolysins was assessed on blood agar medium (Columbia agar + 5% horse-blood, Oxoid, Basingstoke, UK) after incubation at 30°C for 18 h. HBL activity was visualized by seeding bacteria onto sheep blood agar (Columbia agar + 5% sheep-blood, Oxoid) [41]. The production of phosphatidylcholine specific phospholipase-C (PC-PLC) was measured by a gel-diffusion assay with a gel containing crude phosphatidylcholine as previously described [41]. Protease secretion was assessed on agar plate containing 1.5% skim-milk [42] after incubation at 37°C for 18 h. For the detection of *plcA*, *sph*, *cytK*, *nheA*, *nheB*, and *nheC* genes, PCR amplification was performed on genomic DNA as previously described [43] and amplicons identified by DNA sequencing.

### Statistical analysis

Comparison of API 50 CHB with the Bruker Biotyper for the identification of species was made using the Pearson’s chi-squared test. P values of ≤ 0.05 were considered statistically significant.

## Results

### Strain collection and identification

In this study, 75 strains belonging to the genus *Bacillus* or *Paenibacillus* were collected. Fifty isolates derived from deep, usually sterile, body sites, such as blood (n = 21), bile (n = 7), drainage (n = 14), urine (n = 3), central venous catheter (n = 2), cranial ventricular catheter (n = 1), sperm (n = 1), and bronchial aspirate (n = 1). Twenty-five isolates were collected from nails (n = 2), sputum (n = 1), as well as from cutaneous (n = 10), ear (n = 4), nasal (n = 5), pharyngeal (n = 1), ocular (n = 1) or oral (n = 1) swabs (S1 Table).

All strains were subjected to biochemical identification by the API 50 CHB and identification by MALDI-TOF MS. Sequencing of the 16S rRNA gene, the reference technique to identify clinical isolates, was applied to solve identification discrepancies or failures with one of the other methods. The five strains that were identified by 16S rDNA sequencing as *B*. *cereus*/*B*. *thuringiensis* were also subjected to sequencing of the *rpoB* gene [35]. BLAST analysis of these sequences revealed a > 98% identity with either *B*. *cereus* or *B*. *thuringiensis* sequences present in the database. Therefore, as already demonstrated for these two species [35, 44], our analysis confirmed that the five isolates belonged to the *B*. *cereus*/*B*. *thuringiensis* clade. Parasporal crystals, swimming and growth behavior were analyzed for the taxonomic resolution of all the isolates belonging to *B*. *cereus sensu latu* group [22, 23]. All strains (n = 28) did not produce *B*. *thuringiensis*-specific parasporal crystals and three of them were identified as *B*. *mycoides* for their typical rhizoidal growth and inability to swim [34]. In S1 Table, the collected strains are divided based on their final identification.

All *B*. *cereus* strains (n = 25) were correctly identified by MALDI-TOF MS. Six of these strains were misidentified and three unidentified by the API 50 CHB tests. The Bruker Biotyper MS system also allowed the identification of all *B*. *mycoides* (n = 3) isolates, while API 50 CHB misidentified one of these strains.

Biochemical testing misidentified three strains among the 11 *B*. *subtilis* isolates. Three *B*. *subtilis* strains were unidentified by MS. All *B*. *pumilus* (n = 14), *B*. *megaterium* (n = 5), and *B*. *flexus* (n = 3) strains were correctly identified by MS. One *B*. *pumilus*, one *B*. *megaterium*, and all *B*. *flexus* strains were misidentified by biochemical testing. Two out of the five *B*. *licheniformis* isolates were correctly identified by biochemical or MS analysis. However, two strains were misidentified by the API 50 CHB system and no misidentification was obtained by the use of MALDI-TOF MS. All *B*. *simplex* (n = 5) and *Paenibacillus* (n = 4) isolates were misidentified by biochemical testing. The *B*. *simplex* strains, one *Paenibacillus glucanolyticus*, one *Paenibacillus amylolyticus*, and one *Paenibacillus lautus* were unidentified by MALDI-TOF MS. The Bruker Biotyper identified one *B*. *simplex* and one *P*. *amylolyticus* at the genus level.

The frequency of correct species identification by biochemical API 50 CHB analysis and by MALDI-TOF MS is shown in Table 1. The overall performance of MALDI-TOF MS was significantly better (P = 0.004) than that of API 50 CHB for the identification of the species in our collection of isolates (81.3% and 60.0% respectively).

**Table 1:** Percentage of bacteria correctly identified to the species level by API 50 CHB and MALDI-TOF MS.

Species(n. of strains)	API 50 CHB(n. of strains)	API 50 CHB(n. of unidentified/misidentified strains)	MALDI-TOF MS(n. of strains)	MALDI-TOF MS(n. of unidentified-misidentified strains)
*B*. *cereus* (25)	64.0 (16)	(3–6)	100 (25)	(0–0)
*B*. *pumilus* (14)	92.8 (13)	(0–1)	100 (14)	(0–0)
*B*. *subtilis* (11)	72.7 (8)	(0–3)	72.7 (8)	(3–0)
*B*. *licheniformis* (5)	40.0 (2)	(1–2)	40.0 (2)	(3–0)
*B*. *megaterium* (5)	80.0 (4)	(0–1)	100 (5)	(0–0)
*B*. *simplex* (5)	0.0 (0)	(0–5)	0.0 (0)	(5–0)
*B*. *mycoides* (3)	66.7 (2)	(1–0)	100 (3)	(0–0)
*B*. *flexus* (3)	0.0 (0)	(0–3)	100 (3)	(0–0)
*P*. *glucanolyticus* (2)	0.0 (0)	(0–2)	50.0 (1)	(1–0)
*P*. *amylolyticus* (1)	0.0 (0)	(0–1)	0.0 (0)	(1–0)
*P*. *lautus* (1)	0.0 (0)	(0–1)	0.0 (0)	(1–0)
Total (75)	60.0 (45)		81.3 (61)	

### Prevalence and antimicrobial susceptibility

One third of the isolates resulted to belong to the *B*. *cereus* species (Table 2). Interestingly, also *B*. *pumilus* and *B*. *subtilis* were isolated at high rate (18.7% and 14.7%, respectively). While *B*. *cereus* was isolated at similar rates from deep, usually sterile body sites and from superficial and/non-sterile body sites, most of *B*. *pumilus* isolates derived from deep sites (11/14 strains) and accounted for 22% of the total isolations from these samples, while *B*. *subtilis* accounted for 24% of the total isolations from superficial sites. The frequency of the other species from total samples was less than 7%. However, *B*. *licheniformis* and *Paenibacillus* spp. strains were only isolated from sterile body sites and accounted for 10% and 8% of the total isolates from these districts, respectively (4% *P*. *glucanolyticus*, 2% *P*. *amylolyticus*, 2% *P*. *lautus*) (Table 2).

**Table 2:** Frequency of isolation of *Bacillus* and *Paenibacillus* species from deep, usually sterile body sites or superficial and/or non-sterile body sites.

Species	Total samples(n. of strains)	Deep body sites(n. of strains)	Superficial body sites(n. of strains)
*B*. *cereus*	33.3% (25)	32.0% (16)	36.0% (9)
*B*. *pumilus*	18.7% (14)	22.0% (11)	12.0% (3)
*B*. *subtilis*	14.7% (11)	10.0% (5)	24.0% (6)
*B*. *licheniformis*	6.7% (5)	10.0% (5)	0.0% (0)
*B*. *megaterium*	6.7% (5)	6.0% (3)	8.0% (2)
*B*. *simplex*	6.7% (5)	6.0% (3)	8.0% (2)
*B*. *mycoides*	4.0% (3)	2.0% (1)	8.0% (2)
*B*. *flexus*	4.0% (3)	4.0% (2)	4.0% (1)
*P*. *glucanolyticus*	2.7% (2)	4.0% (2)	0.0% (0)
*P*. *amylolyticus*	1.3% (1)	2.0% (1)	0.0% (0)
*P*. *lautus*	1.3% (1)	2.0% (1)	0.0% (0)
Total	100% (75)	100% (50)	100% (25)

The results of the E-test assay expressed as MIC range, MIC 90%, MIC 50% for the 75 isolates are shown in Table 3. All *B*. *cereus*, *B*. *licheniformis*, *B*. *simplex*, *B*. *mycoides*, *P*. *glucanolyticus*, and *P*. *lautus* strains resulted to be resistant to PEN. All the other species were susceptible to this antibiotic. All bacteria were also susceptible to CIP, TET, TGC, and VCM.

**Table 3:** MICs (mg/L) of penicillin, ciprofloxacin, tetracycline, tigecycline, and vancomycin against clinical *Bacillus* and *Paenibacillus* isolates.

Species(n. of strains)	PEN			CIP			TET			TGC			VCM		
	range*	50%^a^	90%^a^	range	50%	90%	range	50%	90%	range	50%	90%	range	50%	90%
*B*. *cereus* (25)	8 to >256	>256	>256	0.064–0.25	0.125	0.19	0.047–4	0.5	4	0.094–0.5	0.25	0.5	0.094–4	0.75	1
*B*. *pumilus* (14)	0.008–0.094	0.032	0.064	0.047–0.15	0.064	0.125	0.25–1	0.38	0.5	0.19–0.5	0.38	0.5	0.094–0.19	0.125	0.125
*B*. *subtilis* (11)	0.008–0.032	0.016	0.032	0.047–0.094	0.064	0.064	0.125–4	2	3	0.19–0.5	0.38	0.5	0.094–0.75	0.125	0.25
*B*. *licheniformis* (5)	0.25 to >256	>256	>256	0.047–0.064	0.047	0.064	0.125–2	0.19	2	0.094–0.5	0.38	0.5	0.19–0.38	0.19	0.25
*B*. *megaterium* (5)	0.047–0.125	0.064	0.125	0.047–0.125	0.094	0.125	0.19–0.38	0.25	0.38	0.19–0.5	0.38	0.5	0.094–0.19	0.094	0.19
*B*. *simplex* (5)	0.25–0.5	0.25	0.5	0.125–0.19	0.125	0.19	0.094–0.125	0.125	0.125	0.094–0.5	0.38	0.5	0.094–0.5	0.19	0.5
*B*. *mycoides* (3)	16 to >256	>256		0.064	0.064		0.38–0.5	0.5		0.19–0.5	0.5		0.75	0.75	
*B*. *flexus* (3)	0.023–0.125	0.125		0.064–0.125	0.064		0.094–0.19	0.125		0.5	0.5		0.094	0.094	
*P*. *glucanolyticus* (2)	4			0.064–0.125			0.5–4			0.016–0.032			0.5	0.5	
*P*. *amylolyticus* (1)	0.047			0.032			0.19			0.032			0.125	0.125	
*P*. *lautus* (1)	32			0.19			4			0.19			0.5	0.5	

* Maximal and minimal MIC values.

^a^ MIC at which 50% or 90% of tested isolates are inhibited. The used interpretative criteria were S: ≤0.12, R: ≥ 0.25 for PEN; S: ≤ 1.0, R: ≥ 4 for CIP; S: ≤ 4, I: = 8, R: ≥ 16 for TET; S: ≤ 0.5 for TGC, and S: ≤ 4 for VCM.

### Pathogenic potential

Hemolysis on blood agar emerged with all *B*. *cereus* and 92.8% of the *B*. *pumilus* isolates (Table 4). HBL and PC-PLC secretion were typical features of *B*. *cereus* strains, being the first toxin produced by 84% and the second by 88% of the *B*. *cereus* isolates. In contrast, most of the species produced extracellular proteases, with the exception of *B*. *simplex*, *P*. *glucanolyticus* and *P*. *lautus* (Table 4). In particular, protease secretion was revealed for all *B*. *pumilus*, *B*. *subtilis*, *B*. *megaterium*, *B*. *flexus*, and *P*. *amylolyticus* strains, while different frequencies of producers were found in *B*. *licheniformis* (60%) and *B*. *mycoides* (33.3%).

**Table 4:** Percentages of strains among clinical *Bacillus* and *Paenibacillus* isolates producing extracellular toxins/enzymes or possessing virulence genes.

Species(n. of strains)	Toxins/enzymes				Virulence genes					
	Hemolysins	HBL	PC-PLC	Proteases	*plca*	*sph*	*cytK*	*nheA*	*nheB*	*nheC*
*B*. *cereus* (25)	100	84.0	88.0	56.0	40.40.0	52.0	24.0	60.0	56.0	56.0
*B*. *pumilus* (14)	92.8	0.0	0.0	100	0.0	0.0	0.0	71.4	50.8	57.1
*B*. *subtilis* (11)	0.0	0.0	0.0	100	0.0	0.0	0.0	18.2	0.0	0.0
*B*. *licheniformis* (5)	0.0	0.0	0.0	60.0	0.0	0.0	0.0	0.0	0.0	0.0
*B*. *megaterium* (5)	0.0	0.0	0.0	100	0.0	0.0	0.0	20.0	0.0	0.0
*B*. *simplex* (5)	0.0	0.0	0.0	0.0	0.0	0.0	0.0	0.0	0.0	0.0
*B*. *mycoides* (3)	0.0	0.0	0.0	33.3	0.0	0.0	0.0	0.0	0.0	0.0
*B*. *flexus* (3)	0.0	0.0	0.0	100	0.0	0.0	0.0	33.3	0.0	33.3
*P*. *glucanolyticus* (2)	0.0	0.0	0.0	0.0	0.0	0.0	0.0	0.0	0.0	0.0
*P*. *amylolyticus* (1)	0.0	0.0	0.0	100	0.0	0.0	0.0	100	0.0	100
*P*. *lautus* (1)	0.0	0.0	0.0	0.0	0.0	0.0	0.0	0.0	0.0	0.0
Total (75)	50.6	28.0	29.3	69.3	13.3	17.3	8.0	40.0	28.0	32.0

The presence of the *plcA*, *sph*, *cytK*, as well as *nheA*, *nheB* and *nheC* genes encoding the phosphatidylinositole-specific phospholipase C, sphingomyelinase, CytK, and the three components of NHE, respectively, was evaluated by PCR amplification with available specific primers [43]. While *plcA*, *sph*, and *cytK* genes were only detected in *B*. *cereus* strains, *nheA*, *nheB* or *nheC* sequences were found in *B*. *cereus*, *B*. *pumilus*, *B*. *subtilis*, *B*. *megaterium*, *B*. *flexus* and *P*. *amylolyticus* (Table 4). However, 10 *B*. *cereus* and four *B*. *pumilus* strains only (representing 40.0% and 28.6% for each species respectively) contained the three genes encoding the entire toxic complex.

Swimming and swarming motility assays were performed with all the isolates. With the exclusion of *B*. *simplex* and *B*. *mycoides*, most of the strains belonging to the other species were motile in the swimming assays (Table 5). The swarming motility assays were performed in culture conditions proved efficient for *B*. *cereus* and *B*. *subtilis* [38, 39]. A high percentage (from 76.0% to 100%) of the *B*. *cereus*, *B*. *pumilus*, *B*. *subtilis*, *B*. *licheniformis*, *P*. *glucanolyticus*, and *P*. *lautus* isolates were able to swarm. In agreement with the absence of swimming motility that is required for swarming [45], the *B*. *simplex* and *B*. *mycoides* isolates did not swarm on the agar plates. No swarming migration was also shown for *B*. *megaterium*, *B*. *flexus* and *P*. *amylolyticus* strains in the used culture conditions (Table 5).

**Table 5:** Percentages of strains among clinical *Bacillus* and *Paenibacillus* isolates showing virulence-related behaviors.

Species(n. of strains)	Swimming	Swarming	Biofilm*
*B*. *cereus* (25)	88.0	76.0	96.0
*B*. *pumilus* (14)	100	93.0	100
*B*. *subtilis* (11)	100	100	100
*B*. *licheniformis* (5)	80.0	80.0	100
*B*. *megaterium* (5)	100	0.0	40.0
*B*. *simplex* (5)	0.0	0.0	60.0
*B*. *mycoides* (3)	0.0	0.0	100
*B*. *flexus* (3)	66.6	0.0	100
*P*. *glucanolyticus* (2)	100	100	100
*P*. *amylolyticus* (1)	100	0.0	0.0
*P*. *lautus* (1)	100	100	100
Total (75)	82.6	6.6	90.6

*Biofilm production as resulting from evaluation in LB or EPS media.

Since nutrient availability is one of the major factors affecting biofilm formation, we first analyzed the ability of the collected strains to develop biofilms in rich medium (LB) and, if strains were not able to form biofilm in this condition, they were retested in low-nutrient medium (EPS) [40]. All *B*. *pumilus*, *B*. *subtilis*, *B*. *licheniformis*, *B*. *mycoides*, *B*. *flexus*, *P*. *glucanolyticus*, and *P*. *lautus* isolates produced biofilm in at least one of the tested conditions. With the exception of the single *P*. *amylolyticus* isolate, the remaining species contained variable amounts of strains (from 96% of *B*. *cereus* to 40% of *B*. *megaterium*) able to form biofilm communities (Table 5 and S1 Table).

## Discussion

Conventional methods based on biochemical and phenotypic techniques for the identification of aerobic Gram-positive spore bearing bacilli still prevail in the clinical microbiology laboratory. However, due to the similarities among closely related species, species identification is sometimes difficult. 16S rRNA gene sequencing remains the gold standard approach in many cases, although it is not always practical for routine use due to its high cost and burden on laboratory technicians. In the last years, MALDI-TOF MS has emerged as rapid, reliable diagnostic tool for the identification of most microorganisms [46] and several platforms and databases have been developed, including the Bruker Biotyper. Phyloproteomic analysis by MALDI-TOF MS has been shown to provide more information on the classification of strains within the *Bacillus* genus compared to 16S rDNA sequencing [28] and its accuracy in *Bacillus* and *Paenibacillus* spp. identification has been proven [5, 24–30]. In our study, the Biotyper correctly identified 61 out of the 75 clinical *Bacillus* and *Paenibacillus* isolates, while API 50 CHB only identified 45 of them. By using the Biotyper, no misidentification was obtained. While all *B*. *cereus*, *B*. *mycoides*, *B*. *pumilus*, and *B*. *megaterium* strains were correctly identified by our MALDI-TOF MS system, this method was unable to identify *B*. *simplex* and most *Paenibacillus* strains. We can hypothesize that this identification failure is due to the limited number of strains (one or two) of *B*. *simplex*, *P*. *glucanolyticus*, *P*. *amylolyticus*, and *P*. *lautus* included in the MALDI-TOF database. In fact, commercial databases are mostly designed for the identification of species that are encountered at higher frequency in clinical practice [27]. To overcome this limitation, a new platform using open reference databases for microorganism identification has been developed [27].

In this study, despite a standard protein extraction step (formic acid and acetonitrile) was applied, the Biotyper could not identify three out of the 11 *B*. *subtilis* and three out of the five *B*. *licheniformis* isolates too. As previously indicated for many Gram-positive bacteria [46], we cannot exclude that additional and/or alternative pretreatment strategies could help in enhancing lysis of the thick peptidoglycan layer these bacteria possess, thus leading to better MALDI-TOF MS results.

Within the *Bacillus* and *Paenibacillus* genera, *B*. *anthracis* and *B*. *cereus* are the predominant pathogens of medical importance, although few *B*. *anthracis* isolations are currently reported. In this study, *B*. *cereus* emerged as the species most frequently isolated from either deep sterile or superficial non-sterile body sites (Table 2). This finding correlates with the role of this bacterium as pathogen/opportunistic pathogen in local and systemic infections in humans [3]. *B*. *pumilus* was also frequently isolated, particularly from sterile body sites. Although known for its plant growth promoting activity, reports of infections due to *B*. *pumilus* are becoming more frequent in the literature [8].

According to its wide distribution throughout the environment, particularly in soil, air, and decomposing plant residue, *B*. *subtilis* was predominantly isolated from non-sterile body sites. This species was isolated from sterile body sites at an equal or comparable frequency to that of *B*. *licheniformis*, *B*. *megaterium* and *B*. *simplex*. Due to the lack of information about the clinical history of the patients from which these bacteria were isolated, we can only speculate that the immune state of these patients and/or long-term indwelling foreign bodies, such as catheters, could have favored the entrance and maintaining of these bacteria in deep body sites.

For the treatment of infections caused by *Bacillus* or *Paenibacillus* strains, little advice is available. We found that all *B*. *cereus*, *B*. *licheniformis*, *B*. *simplex*, *B*. *mycoides* and most *Paenibacillus* isolates are resistant to PEN, whereas the other species are susceptible to this antibiotic. Resistance of *B*. *cereus* and *B*. *mycoides* to PEN was previously reported [37]. Our data indicate that the susceptibility of *B*. *cereus* and *B*. *mycoides* towards CIP, TET, TGC, and VCM, already shown by Luna and coworkers [37], is actually widespread in the *Bacillus* genus. These results are in agreement with the effective eradication of *Bacillus* or *Paenibacillus* bacteria from deep infections, which is usually obtained in our hospital following the treatment with TET or VCM.

As expected, many of the *B*. *cereus* isolates in our collection synthesize a variety of virulence proteins, in particular hemolysins, HBL, PC-PLC, and proteases and possess the *sph*, *plcA*, and the three NHE encoding genes. Moreover, we found a 24% positivity for the *cytK* gene. The finding that the frequency of strains containing *cytK* is lower than usually reported for this gene [47, 48] can be due to the fact that our collection includes clinical isolates and no food poisoning or food related strain.

Few data are available on the production of *B*. *cereus*-like toxins by *Bacillus* species outside the *B*. *cereus sensu lato* group. Even if the genes encoding HBL were sporadically found in *B*. *licheniformis*, *B*. *simplex*, *B*. *megaterium*, and *B*. *subtilis* [18, 19], in this study no HBL activity was detected for isolates outside the *B*. *cereus* species. In agreement with the ability of *B*. *pumilus* to secrete hemolysins and proteases [49], we found that all our *B*. *pumilus* isolates are proteolytic, most of them are hemolytic, but no strain is able to produce PC-PLC. In addition, the finding that most of the strains in our collection is able to secrete extracellular proteases correlates with the notion that *Bacillus* species are among the most prominent groups of extracellular protease producers in bacteria. Secreted proteases, that are essential for the proliferation and growth of bacteria, can degrade host-associated proteins, thereby playing a direct role in bacterial virulence.

The presence of *B*. *cereus*-like toxin genes was previously found in *Bacillus* species outside the *B*. *cereus sensu latu* group. Sequences of the NHE encoding genes were detected in *B*. *licheniformis*, *B*. *simplex*, *B*. *subtilis*, and *Paenibacillus* spp., and *cytK* sequences were found in *B*. *licheniformis*, *B*. *simplex* and *B*. *subtilis* [18]. In this study, we detected the presence of the three genes encoding NHE in four *B*. *pumilus* strains and sequences of *nheA* and/or *nheC* were found in *B*. *pumilus*, *B*. *subtilis*, *B*. *megaterium*, *B*. *flexus*, and *P*. *amylolyticus*. Gene transfer or evolutionary mechanisms could explain the presence of such sequences in non-*B*. *cereus* species.

Flagellum-driven bacterial motility, such as swimming or swarming, may facilitate the invasion of human and nonhuman host cellular barriers [45]. As previously reported for *B*. *cereus* [13, 38], the high frequency of swimming- (82.6%) and swarming-proficient (66.6%) isolates in our collection suggests that these behaviors contribute to the capacity of these strains to colonize and potentially establish an infection in humans. In addition, this is the first report in which this kind of motility has been demonstrated for *B*. *pumilus*, *B*. *licheniformis*, and *P*. *glucanolyticus*.

Biofilm formation is a microbial strategy allowing cells to survive in hostile conditions and providing resistance to natural host defenses. Biofilms are associated with an increased capacity of bacteria to survive within hospital environments and on implanted medical devices [50]. Biofilms can also facilitate wound chronicity and persistence by creating a barrier against neutrophils, macrophages, and antimicrobials [51]. In this study, we demonstrate that biofilm formation is a widespread virulence behavior of clinical *Bacillus* and *Paenibacillus* isolates. This result highlights the importance of catheter removal, commonly practiced in our hospital, in the case of deep *Bacillus* or *Paenibacillus* infections.

In conclusion, the isolation of bacteria belonging to the *Bacillus* or *Paenibacillus* genera should not be disregarded and their identification performed, particularly when samples derive from patients in any preexisting disease condition or immunocompromised. In fact, despite these bacteria are commonly considered soil-related organisms, they are increasingly isolated from hospitalized patients and appear sufficiently equipped of virulence properties that can allow them to behave as pathogens/opportunistic pathogens for humans.

## Supporting Information

S1 Table Contains details regarding identification and virulence potential of each isolate. (DOCX)

## References

[1] Ash C, Priest FG, Collins MD. Molecular identification of rRNA group 3 bacilli (Ash, Farrow, Wallbanks and Collins) using a PCR probe test. Proposal for the creation of a new genus *Paenibacillus*. Antonie Van Leeuwenhoek. 1993–1994;64(3–4):253–60. 10.1007/BF00873085 8085788

[2] Brown KL. Control of bacterial spores. Br Med Bull 2000;56(1):158–171. 10885113 10.1258/0007142001902860

[3] Jeßberger N, Krey VM, Rademacher C, Böhm ME, Mohr A-K, Ehling-Schulz M, et al From genome to toxicity: a combinatory approach highlights the complexity of enterotoxin production in *Bacillus cereus*. Front Microbiol. 2015 6 10;6:560 10.3389/fmicb.2015.00560 26113843 PMC4462024

[4] Abrishami M, Hashemi B, Abrishami M, Abnous K, Razavi-Azarkhiavi K, Behravan J. PCR detection and identification of bacterial contaminants in ocular samples from post-operative endophthalmitis. J Clin Diagn Res. 2015 4 9(4):NC01–NC03. 10.7860/JCDR/2015/10291.5733 26023576 PMC4437090

[5] Ferrand J, Hadou T, Selton-Suty C, Goehringer F, Sadoul N, Alauzet C, et al Cardiac device-related endocarditis caused by *Paenibacillus glucanolyticus*. J Clin Microbiol. 2013 10;51(10):3439–42. 10.1128/JCM.00864-13 23884996 PMC3811670

[6] Idelevich EA, Pogoda CA, Ballhausen B, Wüllenweber J, Eckardt L, Baumgartner H, et al Pacemaker lead infection and related bacteraemia caused by normal and small colony variant phenotypes of *Bacillus licheniformis*. J Med Microbiol. 2013 6;(Pt 6);62:940–4. 10.1099/jmm.0.051987-0 23518654

[7] Jeon YL, Yang JJ, Kim MJ, Lim G, Cho SY, Park TS, et al Combined *Bacillus licheniformis* and *Bacillus subtilis* infection in a patient with esophageal perforation. J Med Microbiol. 2012 12;61(Pt 12):1766–9. 10.1099/jmm.0.042275-0 22918867

[8] Kimouli M, Vrioni G, Papadopoulou M, Koumaki V, Petropoulou D, Gounaris A, et al Two cases of severe sepsis caused by *Bacillus pumilus* in neonatal infants. J Med Microbiol. 2012 4;61(Pt4):596–9. 22174371 10.1099/jmm.0.033175-0

[9] Padhi S, Dash M, Sahu R, Panda P. Urinary tract infection due to *Paenibacillus alvei* in a chronic kidney disease: a rare case report. J Lab Physicians. 2013 7;5(2):133–5. 10.4103/0974-2727.119872 24701110 PMC3968626

[10] Rieg S, Martin Bauer T, Peyerl-Hoffmann G, Held J, Ritter W, Wagner D, et al *Paenibacillus larvae* bacteremia in injection drug users. Emerg Infect Dis. 2010 3 16(3):487–9. 10.3201/eid1603.091457 20202425 PMC3322038

[11] Sahu C, Kumar K, Sinha MK, Venkata A, Majji AB, Jalali S. Review of endogenous endophthalmitis during pregnancy including case series. Int Ophthalmol. 2013 10;33(5):611–8. 10.1007/s10792-012-9697-z 23263732

[12] Senesi S, Ghelardi E. Production, secretion and biological activity of *Bacillus cereus* enterotoxins. Toxins (Basel) 2010 7;2(7):1690–703. 22069656 10.3390/toxins2071690 PMC3153264

[13] Senesi S, Salvetti S, Celandroni F, Ghelardi E. 2010. Features of *Bacillus cereus* swarm cells. Res Microbiol. 2010 11;161(9):743–9. 10.1016/j.resmic.2010.10.007 21035546

[14] Celandroni F, Salvetti S, Senesi S, Ghelardi E. *Bacillus thuringiensis* membrane-damaging toxins acting on mammalian cells. FEMS Microbiol Lett. 2014 12;361(2):95–103 10.1111/1574-6968.12615 25283838

[15] Ramarao N, Sanchis V. The pore-forming haemolysins of *Bacillus cereus*: a review. Toxins. 2013 6;5(6):1119–39. 10.3390/toxins5061119 23748204 PMC3717773

[16] Funada H, Uotani C, Machi T, Matsuda T, Nonomura A. *Bacillus cereus* bacteremia in an adult with acute leukemia. Jpn J Clin Oncol 1988 3;18(1):69–74. 3127617 10.1093/jjco/18.1.69

[17] Kuroki R, Kawakami K, Qin L, Kaji C, Watanabe K, Kimura Y, et al Nosocomial bacteremia caused by biofilm-forming *Bacillus cereus* and *Bacillus thuringiensis*. Intern Med. 2009 5;48(10):791–6. 19443973 10.2169/internalmedicine.48.1885

[18] De Bellis P, Minervini F, Di Biase M, Valerio F, Lavermicocca P, Sisto A. Toxigenic potential and heat survival of spore-forming bacteria isolated from bread and ingredients. Int J Food Microbiol. 2015 3;197(16):30–9. 25555227 10.1016/j.ijfoodmicro.2014.12.017

[19] Rowan NJ, Deans K, Anderson JG, Gemmell CG, Hunter IS, Chaithong T. Putative virulence factor expression by clinical and food isolates of *Bacillus* spp. after growth in reconstituted infant milk formulae. Appl Environ Microbiol. 2001 9;67(9):3873–81. 11525980 10.1128/AEM.67.9.3873-3881.2001 PMC93104

[20] Ameur MA, Dubrous P, Koeck JL. *Bacillus licheniformis*: an unusual cause of erysipelosis. Med Mal Infect. 2005 Jul-Aug;35(7–8):417–8. 15982842 10.1016/j.medmal.2005.04.007

[21] Kumar S, Shah AK. Characterization of an adhesive molecule from *Bacillus megaterium* ADE-0-1. Carbohydr Polym. 2015 3 6;117:543–8. 10.1016/j.carbpol.2014.10.017 25498669

[22] Tourasse NJ, Helgason E, Klevan A, Sylvestre P, Moya M, Haustant M, et al Extended and global phylogenetic view of the *Bacillus cereus* group population by combination of MLST, AFLP, and MLEE genotyping data. Food Microbiol. 2011 4;28(2):236–44. 10.1016/j.fm.2010.06.014 21315979

[23] Rasko DA, Altherr MR, Han CS, Ravel J. Genomics of the *Bacillus cereus* group of organisms. FEMS Microbiol Rev. 2005 4;29(2):303–29. 15808746 10.1016/j.femsre.2004.12.005

[24] AlMasoud N, Xu Y, Nicolaou N, Goodacre R. Optimization of matrix assisted desorption/ionization time of flight mass spectrometry (MALDI-TOF-MS) for the characterization of *Bacillus* and *Brevibacillus* species. Anal Chim Acta. 2014 8 20;840:49–57. 10.1016/j.aca.2014.06.032 25086893 PMC4223412

[25] Böhme K, Fernández-No IC, Barros-Velázquez J, Gallardo JM, Cañas B, Calo-Mata P. Rapid species identification of seafood spoilage and pathogenic Gram-positive bacteria by MALDI-TOF mass fingerprinting. Electrophoresis. 2011, 11;32(21):2951–65. 10.1002/elps.201100217 22009363

[26] Farfour E, Leto J, Barritault M, Barberis C, Meyer J, Dauphin B, et al Evaluation of the Andromas matrix-assisted laser desorption ionization-time of flight mass spectrometry system for identification of aerobically growing Gram-positive bacilli. J Clin Microbiol. 2012 8;50(8):2702–7. 10.1128/JCM.00368-12 22692743 PMC3421524

[27] Starostin KV, Demidov EA, Bryanskaya AV, Efimov VM, Rozanov AS, Peltek SE. Identification of *Bacillus* strains by MALDI TOF MS using geometric approach. Sci Rep. 2015 11 23;5:16989 10.1038/srep16989 26592761 PMC4655313

[28] Fernández-No IC, Böhme K, Díaz-Bao M, Cepeda A, Barros-Velázquez J, Calo-Mata P. Characterisation and profiling of *Bacillus subtilis*, *Bacillus cereus* and *Bacillus licheniformis* by MALDI-TOF mass fingerprinting. Food Microbiol 2013 4;33(2):235–42. 10.1016/j.fm.2012.09.022 23200657

[29] Hotta Y, Sato J, Sato H, Hosoda A, Tamura H. Classification of the genus *Bacillus* based on MALDI-TOF MS analysis of ribosomal proteins coded in S10 and spc operons. J Agric Food Chem. 2011 5 25;59(10):5222–30. 10.1021/jf2004095 21469741

[30] Schäfer MO, Genersch E, Fünfhaus A, Poppinga L, Formella N, Bettin B, et al Rapid identification of differentially virulent genotypes of *Paenibacillus larvae*, the causative organism of American foulbrood of honey bees, by whole cell MALDI-TOF mass spectrometry. Vet Microbiol. 2014 6 4;170(3–4):291–7. 10.1016/j.vetmic.2014.02.006 24613082

[31] Celandroni F, Ghelardi E, Pastore M, Lupetti A, Kolstø AB, Senesi S. Characterization of the chemotaxis *fliY* and *cheA* genes in *Bacillus cereus*. FEMS Microbiol Lett. 2000 9 15;190(2):247–53. 11034287 10.1111/j.1574-6968.2000.tb09294.x

[32] Clinical and Laboratory Standards Institute. Interpretive Criteria for Identification of Bacteria and Fungi by DNA Target Sequencing Approved Standard MM18-A. 2008 CLSI, Wayne, PA.

[33] Ghelardi E, Celandroni F, Salvetti S, Fiscarelli E, Senesi S. *Bacillus thuringiensis* pulmonary infection: critical role for bacterial membrane-damaging toxins and host neutrophils. Microbes Infect. 2007 4;9(5):591–8. 17387030 10.1016/j.micinf.2007.02.001

[34] Di Franco C, Beccari E, Santini T, Pisaneschi G, Tecce G. Colony shape as a genetic trait in the pattern-forming *Bacillus mycoides*. BMC Microbiol 2002 11 13;2:33 12429070 10.1186/1471-2180-2-33 PMC138795

[35] Ki JS, Zhang W, Qian PY. Discovery of marine *Bacillus* species by 16S rRNA and *rpoB* comparisons and their usefulness for species identification. J Microbiol Methods 2009 4;77(1):48–57. 10.1016/j.mimet.2009.01.003 19166882

[36] Clinical and Laboratory Standards Institute. Methods for Antimicrobial Dilution and Disk Susceptibility Testing of Infrequently Isolated or Fastidious Bacteria: Approved guideline M45-A2. 2010 CLSI, Wayne, PA.

[37] Luna VA, King DS, Gulledge J, Cannons AC, Amuso PT, Cattani J. Susceptibility of *Bacillus anthracis*, *Bacillus cereus*, *Bacillus mycoides*, *Bacillus pseudomycoides* and *Bacillus thuringiensis* to 24 antimicrobials using Sensititre automated microbroth dilution and Etest agar gradient diffusion methods. J Antimicrob Chemother. 2007 9 60(3):555–67. 17586563 10.1093/jac/dkm213

[38] Ghelardi E, Celandroni F, Salvetti S, Ceragioli M, Beecher DJ, Senesi S, et al Swarming behavior of and hemolysin BL secretion by *Bacillus cereus*. Appl Environ Microbiol. 2007 6 73(12): 4089–93. 17449693 10.1128/AEM.02345-06 PMC1932716

[39] Ghelardi E, Salvetti S, Ceragioli M, Gueye SA, Celandroni F, Senesi S. Contribution of surfactin and SwrA to flagellin expression, swimming, and surface motility in *Bacillus subtilis* Appl Environ Microbiol. 2012 9 78(18):6540–44. 10.1128/AEM.01341-12 22773650 PMC3426715

[40] Hsueh Y-H, Somers EB, Lereclus D, Wong ACL. Biofilm formation by *Bacillus cereus* is influenced by PlcR, a pleiotropic regulator. Appl Environ Microbiol. 2006 7;72(7):5089–92. 16820512 10.1128/AEM.00573-06 PMC1489338

[41] Ghelardi E, Celandroni F, Salvetti S, Beecher DJ, Gominet M, Lereclus D, et al Requirement of *flhA* for swarming differentiation, flagellin export, and secretion of virulence-associated proteins in *Bacillus thuringiensis*. J Bacteriol. 2002 12;184(23):6424–33 12426328 10.1128/JB.184.23.6424-6433.2002 PMC135439

[42] Wandersman C, Andro T, Bertheau Y. Extracellular proteases in *Erwinia chrysanthemi*. J Gen Microbiol. 1986;132:899–906.

[43] Ghelardi E, Celandroni F, Salvetti S, Barsotti C, Baggiani A, Senesi S. Identification and characterization of toxigenic *Bacillus cereus* isolates responsible for two food-poisoning outbreaks. FEMS Microbiol Lett. 2002 2 19;208(1):129–34. 11934506 10.1111/j.1574-6968.2002.tb11072.x

[44] Caamaño-Antelo S, Fernández-No IC, Böhme K, Ezzat-Alnakip M, Quintela-Baluja M, Barros-Velázquez J, et al Genetic discrimination of foodborne pathogenic and spoilage *Bacillus* spp. based on three housekeeping genes. Food Microbiol. 2015 4 46:288–98. 10.1016/j.fm.2014.08.013 25475298

[45] Partridge JD, Harshey RM. Swarming: flexible roaming plans. J Bacteriol. 2013 3;195(5):909–18. 10.1128/JB.02063-12 23264580 PMC3571328

[46] Clark AE, Kaleta EJ, Arora A, Wolk DM. Matrix-assisted laser desorption ionization-time of flight mass spectrometry: a fundamental shift in the routine practice of clinical microbiology. Clin Microbiol Rev. 2013 7;26(3):547–603. 10.1128/CMR.00072-12 23824373 PMC3719498

[47] Castiaux V, Liu X, Delbrassinne L, Mahillon J. Is Cytotoxin K from *Bacillus cereus* a bona fide enterotoxin? Int J Food Microbiol. 2015 10 15;211:79–85 10.1016/j.ijfoodmicro.2015.06.020 26186121

[48] Yim JH, Kim KY, Chon JW, Kim DH, Kim HS, Choi DS, et al Incidence, Antibiotic Susceptibility, and Toxin Profiles of *Bacillus cereus* sensu lato isolated from Korean Fermented Soybean Products. J Food Sci. 2015 6 86;80:M1266–M1270 10.1111/1750-3841.12872 25950845

[49] Hoult B, Tuxford AF. Toxin production by *Bacillus pumilus*. J Clin Pathol. 1991 6;44(6):455–8. 2066422 10.1136/jcp.44.6.455 PMC496823

[50] Donlan RM, Costerton JW. Biofilms: Survival mechanisms of clinically relevant microorganisms. Clin Microbiol Rev. 2002 4;15(2):167–93 11932229 10.1128/CMR.15.2.167-193.2002 PMC118068

[51] Thurlow LR, Hanke ML, Fritz T, Angle A, Aldrich A, Williams SH, et al *Staphylococcus aureus* biofilms prevent macrophage phagocytosis and attenuate inflammation in vivo. J Immunol. 2011 6 1;186(11):6585–96. 10.4049/jimmunol.1002794 21525381 PMC3110737

