# Supplementary material for: Identification and Pathogenic Potential of Clinical Bacillus and Paenibacillus Isolates
Source: PLoS One. 2016 Mar 31;11(3):e0152831. doi: 10.1371/journal.pone.0152831 (PMC4816569; doi:10.1371/journal.pone.0152831)
Supplement: S1 Table — (DOCX) [file pone.0152831.s001.docx]

S1 Table. Identification and virulence potential of each isolate.

|  | **Source** | **16S rDNA**  **sequencing** | **API 50 CH-B** | **MALDI-TOF** | **Hemolysis** | **HBL** | **Proteases** | **PC-PLC** | **Swimming** | **Swarming** | **Biofilm LB** | **Biofilm EPS** |
| --- | --- | --- | --- | --- | --- | --- | --- | --- | --- | --- | --- | --- |
| ***Bacillus cereus*** | | | | | | | | | | | | |
| Ce1 | blood | *B. cereus* | ND | *B. cereus* | **+** | **+** | **-** | **+** | **+** | **+** | **+** |  |
| GPe1 | blood |  | *B. cereus* | *B. cereus* | **+** | **+** | **+** | **+** | **+** | **+** | **-** | **+** |
| GPe2 | blood |  | *B. cereus* | *B. cereus* | **+** | **+** | **+** | **+** | **+** | **+** | **-** | **+** |
| RCe1 | blood |  | *B. cereus* | *B. cereus* | **+** | **+** | **+** | **+** | **+** | **+** | **+** |  |
| Rce2 | blood |  | *B. cereus* | *B. cereus* | **+** | **+** | **+** | **+** | **+** | **+** | **+** |  |
| MXe1 | blood |  | *B. cereus* | *B. cereus* | **+** | **+** | **+** | **+** | **+** | **+** | **+** |  |
| ABe1 | blood |  | *B. cereus* | *B. cereus* | **+** | **+** | **-** | **+** | **+** | **+** | **-** | **+** |
| MAe1 | blood |  | *B. cereus* | *B. cereus* | **+** | **+** | **-** | **+** | **+** | **+** | **+** |  |
| LVe1(2) | blood |  | *B. cereus* | *B. cereus* | **+** | **+** | **-** | **+** | **-** | **-** | **+** |  |
| Topi | blood | *B. cereus* | *B. stearothermophilus* | *B. cereus* | **+** | **+** | **-** | **+** | **+** | **+** | **+** |  |
| MFpp1 | drainage | *B. cereus/*  *B. thuringiensis* | ND | *B. cereus* | + | + | + | + | **-** | **-** | + |  |
| RLd1 | drainage | *B. cereus/*  *B. thuringiensis* | ND | *B. cereus* | **+** | **-** | **+** | **+** | **+** | **+** | **+** |  |
| GGmv1 | drainage | *B. cereus* | *B. mycoides* | *B. cereus* | **+** | **+** | **-** | **-** | **+** | **+** | **+** |  |
| ALmv1 | drainage |  | *B. cereus* | *B. cereus* | **+** | **+** | **-** | **+** | **+** | **+** | **-** | **+** |
| CXb1 | bile |  | *B. cereus* | *B. cereus* | **+** | **+** | **-** | **+** | **-** | **-** | **+** |  |
| Muc1 | urine | *B. cereus/*  *B. thuringiensis* | *B. licheniformis* | *B. cereus* | **+** | **+** | **+** | **-** | **+** | **+** | **-** | **+** |
| Ges1 | sputum |  | *B. cereus* | *B. cereus* | **+** | **+** | **-** | **+** | **+** | **+** | **+** |  |
| CTn1 | nasal mucosa |  | *B. cereus* | *B. cereus* | **+** | **-** | **+** | **+** | **+** | **-** | **+** |  |
| LEtn1 | nasal mucosa | *B. cereus/*  *B. thuringiensis* | *B. mycoides* | *B. cereus* | **+** | **-** | **-** | **-** | **+** | **+** | **+** |  |
| XFta | ear |  | *B. cereus* | *B. cereus* | **+** | **+** | **+** | **+** | **+** | **+** | **-** | **-** |
| VLta1 | ear | *B. cereus* | *B. firmus* | *B. cereus* | **+** | **-** | **+** | **+** | **+** | **-** | **+** |  |
| BCc1 | skin |  | *B. cereus* | *B. cereus* | **+** | **+** | **+** | **+** | **+** | **+** | **-** | **+** |
| CIc1 | skin |  | *B. cereus* | *B. cereus* | **+** | **+** | **+** | **+** | **+** | **+** | **-** | **+** |
| BMin1 | skin | *B. cereus* | *B. mycoides* | *B. cereus* | **+** | **+** | **-** | **+** | **+** | **-** | **-** | **+** |
| GGu1 | nail |  | *B. cereus* | *B. cereus* | **+** | **+** | **+** | **+** | **+** | **+** | **-** | **+** |
| ***Bacillus mycoides*** | | | | | | | | | | | | |
| VRs1 | sperm |  | *B. mycoides* | *B. mycoides* | **-** | **-** | **-** | **-** | **-** | **-** | **-** | **+** |
| AHtn1 | nasal mucosa |  | *B. mycoides* | *B. mycoides* | **-** | **-** | **-** | **-** | **-** | **-** | **-** | **+** |
| SFx1 | nasal mucosa | *B. cereus/*  *B. thuringiensis* | *ND* | *B. mycoides* | **-** | **-** | **+** | **-** | **-** | **-** | **-** | **+** |
| ***Bacillus subtilis*** | | | | | | | | | | | | |
| FXe1 | blood | *B. subtilis* | *B. stearothermophilus* | *ND* | **-** | **-** | **+** | **-** | **+** | **+** | **-** | **+** |
| BRmt1 | drainage |  | *B. subtilis* | *B. subtilis* | **-** | **-** | **+** | **-** | **+** | **+** | **+** |  |
| GGa1 | drainage |  | *B. subtilis* | *B. subtilis* | **-** | **-** | **+** | **-** | **+** | **+** | **+** |  |
| MPmv1 | drainage |  | *B. subtilis* | *B. subtilis* | **-** | **-** | **+** | **-** | **+** | **+** | **+** |  |
| Bcv1 | central venous catheter |  | *B. subtilis* | *B. subtilis* | **-** | **-** | **+** | **-** | **+** | **+** | **+** |  |
| Of1 | pharyngeal mucosa |  | *B. subtilis* | *B. subtilis* | **-** | **-** | **+** | **-** | **+** | **+** | **+** |  |
| SCtl1 | oral mucosa |  | *B. subtilis* | *B. subtilis* | **-** | **-** | **+** | **-** | **+** | **+** | **+** |  |
| LRtn1 | nasal mucosa | *B. subtilis* | *B. stearothermophilus* | *ND* | **-** | **-** | **+** | **-** | **+** | **+** | **+** |  |
| BFto1 | eye |  | *B. subtilis* | *B. subtilis* | **-** | **-** | **+** | **-** | **+** | **+** | **+** |  |
| GFc1 | skin | *B. subtilis* | *B. subtilis* | ND | **-** | **-** | **+** | **-** | **+** | **+** | **+** |  |
| Bt1 | skin | *B. subtilis* | *B. megaterium* | *B. subtilis* | **-** | **-** | **+** | **-** | **+** | **+** | **+** |  |
| ***Bacillus pumilus*** | | | | | | | | | | | | |
| MTe1 | blood |  | *B. pumilus* | *B. pumilus* | **+** | **-** | **+** | **-** | **+** | **+** | **+** |  |
| VXe1 | blood |  | *B. pumilus* | *B. pumilus* | **+** | **-** | **+** | **-** | **+** | **-** | **+** |  |
| LVe1 | blood | *B. pumilus* | *B. stearothermophilus* | *B. pumilus* | **+** | **-** | **+** | **-** | **+** | **+** | **+** |  |
| PXmt1 | drainage |  | *B. pumilus* | *B. pumilus* | **-** | **-** | **+** | **-** | **+** | **+** | **+** |  |
| MAd1 | drainage |  | *B. pumilus* | *B. pumilus* | **+** | **-** | **+** | **-** | **+** | **+** | **+** |  |
| BPb1 | bile |  | *B. pumilus* | *B. pumilus* | **+** | **-** | **+** | **-** | **+** | **+** | **+** |  |
| DAb1 | bile |  | *B. pumilus* | *B. pumilus* | **+** | **-** | **+** | **-** | **+** | **+** | **+** |  |
| GPb1 | bile |  | *B. pumilus* | *B. pumilus* | **+** | **-** | **+** | **-** | **+** | **+** | **+** |  |
| MXb1 | bile |  | *B. pumilus* | *B. pumilus* | **+** | **-** | **+** | **-** | **+** | **+** | **+** |  |
| Db1 | bile |  | *B. pumilus* | *B. pumilus* | **+** | **-** | **+** | **-** | **+** | **+** | **+** |  |
| DFba1 | bronchial aspirate |  | *B. pumilus* | *B. pumilus* | **+** | **-** | **+** | **-** | **+** | **+** | **+** |  |
| Ata1 | ear |  | *B. pumilus* | *B. pumilus* | **+** | **-** | **+** | **-** | **+** | **+** | **+** |  |
| GXtc1 | skin |  | *B. pumilus* | *B. pumilus* | **+** | **-** | **+** | **-** | **+** | **+** | **+** |  |
| Ixtc1 | skin |  | *B. pumilus* | *B. pumilus* | **+** | **-** | **+** | **-** | **+** | **+** | **+** |  |
| ***Bacillus licheniformis*** | | | | | | | | | | | | |
| LIe1 | blood | *B. licheniformis* | *B. licheniformis* | *ND* | **-** | **-** | **-** | **-** | **-** | **-** | **-** | **+** |
| LGd1 | drainage | *B. licheniformis* | *B. circulans* | *B. licheniformis* | **-** | **-** | **+** | **-** | **+** | **+** | **+** |  |
| BGmv1 | drainage | *B. licheniformis* | N.D | *ND* | **-** | **-** | **-** | **-** | **+** | **+** | **+** |  |
| MFb1 | bile |  | *B. licheniformis* | *B. licheniformis* | **-** | **-** | **+** | **-** | **+** | **+** | **+** |  |
| LPcci1 | cranial ventricular catheter | *B. licheniformis* | *B. amyloliquefaciens* | *ND* | **-** | **-** | **+** | **-** | **+** | **+** | **+** |  |
| ***Bacillus megaterium*** | | | | | | | | | | | | |
| Ge1 | blood |  | *B. megaterium* | *B. megaterium* | **-** | **-** | **+** | **-** | **+** | **-** | **-** | **+** |
| MVlc1 | drainage |  | *B. megaterium* | *B. megaterium* | **-** | **-** | **+** | **-** | **+** | **-** | **-** | **-** |
| Pd1 | drainage |  | *B. megaterium* | *B. megaterium* | **-** | **-** | **+** | **-** | **+** | **-** | **-** | **+** |
| TDt1 | skin |  | *B. megaterium* | *B. megaterium* | **-** | **-** | **+** | **-** | **+** | **-** | **-** | **-** |
| LFc1 | skin | *B. megaterium* | *B. stearothermophilus* | *B. megaterium* | **-** | **-** | **+** | **-** | **+** | **-** | **-** | **-** |
| ***Bacillus simplex*** | | | | | | | | | | | | |
| GGe1 | blood | *B. simplex* | *B. firmus* | ND | **-** | **-** | **-** | **-** | **-** | **-** | **-** | **+** |
| NFc1 | central venous catheter | *B. simplex* | *B. firmus* | ND | **-** | **-** | **-** | **-** | **-** | **-** | **-** | **+** |
| BFuc1 | urine | *B. simplex* | *B. firmus* | ND | **-** | **-** | **-** | **-** | **-** | **-** | **-** | **-** |
| Pta1 | ear | *B. simplex* | *B. circulans* | *Bacillus sp.* | **-** | **-** | **-** | **-** | **-** | **-** | **-** | **+** |
| AVu1 | nail | *B. simplex* | *B. firmus* | ND | **-** | **-** | **-** | **-** | **-** | **-** | **-** | **-** |
| ***Bacillus flexus*** | | | | | | | | | | | | |
| TPe1 | blood | *B. flexus* | *B. coagulans* | *B. flexus* | **-** | **-** | **+** | **-** | **+** | **-** | **+** |  |
| ESe1 | blood | *B. flexus* | *B. megaterium* | *B. flexus* | **-** | **-** | **+** | **-** | **+** | **-** | **+** |  |
| FLtas1 | skin | *B. flexus* | *B. coagulans* | *B. flexus* | **-** | **-** | **+** | **-** | **-** | **-** | **+** |  |
| ***Paenibacillus glucanolyticus*** | | | | | | | | | | | | |
| Oe1 | blood | *P. glucanolyticus* | *B. circulans* | ND | **-** | **-** | **-** | **-** | **+** | **+** | **+** |  |
| IGe1 | blood | *P. glucanolyticus* | *B. circulans* | *P. glucanolyticus* | **-** | **-** | **-** | **-** | **+** | **+** | **+** |  |
| ***Paenibacillus amylolyticus*** | | | | | | | | | | | | |
| Bxmt1 | drainage | *P. amylolyticus* | *B. circulans* | *Paenibacillus sp.* | **-** | **-** | **+** | **-** | **+** | **-** | **-** | **-** |
| ***Paenibacillus lautus*** | | | | | | | | | | | | |
| GLuc1 | urine | *P. lautus* | *B. circulans* | ND | **-** | **-** | **-** | **-** | **+** | **+** | **-** | **+** |
